# Multi-Modal Anomaly Detection in Review Texts with Sensor-Derived Metadata Using Instruction-Tuned Transformers

**DOI:** 10.3390/s25227048

**Published:** 2025-11-18

**Authors:** Khaled M. Alhawiti

**Affiliations:** Faculty of Computers and Information Technology, University of Tabuk, Tabuk 47713, Saudi Arabia; khalhawiti@ut.edu.sa

**Keywords:** zero-shot learning, multi-modal fake review detection, instruction-tuned transformers, sensor-derived metadata, IoT-enabled platforms, interpretability

## Abstract

Fake review detection is critical for maintaining trust and ensuring decision reliability across digital marketplaces and IoT-enabled ecosystems. This study presents a zero-shot framework for multi-modal anomaly detection in user reviews, integrating textual and metadata-derived signals through instruction-tuned transformers. The framework integrates three complementary components: language perplexity scoring with FLAN-T5 to capture linguistic irregularities, unsupervised reconstruction via a transformer-based autoencoder to identify structural deviations, and semantic drift analysis to measure contextual misalignment between task-specific and generic embeddings. To enhance applicability in sensor-driven environments, the framework incorporates device-level metadata such as timestamps, product usage logs, and operational signals to enable cross-validation between unstructured text and structured sensor features. A unified anomaly score fusing textual and sensor-informed signals improves robustness under multi-modal detection scenarios, while interpretability is achieved through token-level saliency maps for textual analysis and feature-level attributions for sensor metadata. Experimental evaluations on the Amazon Reviews 2023 dataset, supplemented by metadata-rich sources including the Amazon Review Data 2018 and Historic Amazon Reviews (1996–2014) datasets demonstrate strong zero-shot performance (AUC up to 0.945) and additional few-shot adaptability under limited supervision (AUC > 0.95), maintaining stable precision–recall trade-offs across product domains. The proposed framework provides real-world impact by enabling real-time, multi-modal fake review detection in IoT-driven platforms and smart spaces, supporting consumer trust, automated decision-making, and transparent anomaly detection in sensor-enhanced digital ecosystems.

## 1. Introduction

Anomaly detection is a critical task for safeguarding the integrity and trustworthiness of user-generated content, particularly in digital marketplaces and review-centric platforms [1]. As online reviews increasingly influence consumer decision-making and recommendation systems, identifying abnormal, manipulated, or semantically inconsistent content has become essential. Such anomalies may appear as incoherent text, injected noise, off-topic commentary, or adversarial inputs intended to mislead users or exploit algorithmic models [2,3,4,5,6,7]. Beyond conventional e-commerce settings, similar challenges arise in IoT-driven ecosystems, where user feedback is often accompanied by sensor-derived metadata such as device logs, timestamps, and operational signals. Detecting anomalies in these multi-modal environments is crucial not only for ensuring the authenticity of reviews but also for supporting trust in sensor-enabled platforms, smart devices, and embedded IoT systems that depend on reliable user- and device-generated information [8].

Traditional anomaly detection approaches typically rely on supervised learning models that demand large volumes of annotated data [9,10]. However, in open-domain review environments, obtaining labeled anomalies is both costly and inherently subjective. The vast diversity of linguistic expressions, writing styles, and domain-specific semantics further complicates the annotation process and limits the scalability of supervised techniques. This has spurred growing interest in developing label-efficient, and especially zero-shot, anomaly detection methods that generalize across domains without reliance on annotated training data [11].

Despite the transformative capabilities of large pre-trained language models (PLMs) in natural language processing (NLP), their use in anomaly detection for multi-domain reviews remains underutilized [12,13]. Instruction-tuned models like FLAN-T5 have demonstrated notable zero-shot generalization for classification and reasoning tasks. Yet, their application to unsupervised or label-free anomaly detection, especially in settings marked by linguistic heterogeneity and semantic drift, has not been fully explored. Existing models are often domain-specific, narrowly scoped, or unable to detect higher-order semantic inconsistencies in open-domain review corpora [14].

A novel zero-shot framework is presented for detecting fake reviews across diverse product domains, leveraging the semantic inference capabilities of instruction-tuned transformers. The architecture integrates three complementary components: (i) linguistic scoring based on language perplexity using FLAN-T5, (ii) structural anomaly detection through a transformer-based autoencoder, and (iii) semantic drift measurement between instruction-guided and unsupervised embeddings. To enhance applicability in IoT-enabled platforms, the framework incorporates sensor-derived metadata such as device logs, timestamps, and operational features, allowing cross-validation of anomalies between textual reviews and structured sensor signals. These components are fused into a unified hybrid anomaly score, enabling the detection of both syntactic irregularities and semantic inconsistencies without reliance on labeled data. Unlike earlier works where perplexity, reconstruction, or embedding drift are used in isolation, our framework fuses them through instruction-tuned semantic conditioning, producing a unified anomaly representation that jointly leverages textual fluency, structural regularity, and metadata-based contextual validation. The proposed integration enables domain-agnostic anomaly inference under zero-shot settings.

The framework is empirically evaluated on the large-scale Amazon Reviews 2023 corpus, comprising over 570 million reviews across 33 product categories, as well as metadata-rich sources such as the Historic Amazon Reviews datasets [15,16,17,18]. Results demonstrate strong zero-shot detection capabilities, robust generalization under domain shift, and measurable gains in few-shot settings, with the integration of metadata further improving anomaly resolution in sensor-driven contexts. To support interpretability, token-level saliency attribution is combined with feature-level attributions from sensor metadata, offering transparent explanations for anomaly decisions. By unifying prompt-based semantic inference, unsupervised structural modeling, latent embedding drift analysis, and metadata-driven validation, the proposed method addresses key challenges in scalable, explainable, and IoT-relevant review fraud detection.

This paper makes the following key contributions:

- Development of a multi-modal zero-shot fake review detection framework using instruction-tuned transformers for domain-agnostic anomaly analysis.
- Design of a hybrid anomaly scoring mechanism combining perplexity, reconstruction error, semantic drift, and metadata-informed signals.
- Definition of a semantic drift metric for measuring contextual misalignment, extended with sensor-derived metadata validation.
- Use of prompt ensemble strategies to improve generalization across diverse product domains and IoT-enabled contexts.
- Integration of token-level saliency and metadata-level attribution to ensure transparent and interpretable anomaly detection.
- Empirical validation on Amazon Reviews 2023, Amazon Review Data 2018, and Historic Amazon datasets, achieving up to 0.945 AUC with strong cross-domain generalization.

The remainder of this paper is structured as follows: Section 2 reviews related work; Section 3 presents the proposed methodology; Section 4 describes the dataset and development environment; Section 5 reports the experimental results; and Section 6 concludes with future research directions.

## 2. Related Work

Recent advances in anomaly detection have significantly improved the capacity to identify irregular patterns in both structured and unstructured datasets [19,20,21]. In the context of natural language, particularly user-generated reviews, anomaly detection remains challenging due to high semantic variability, domain heterogeneity, and the scarcity of labeled data. These challenges are further amplified in IoT-driven ecosystems, where textual reviews are often coupled with sensor-derived metadata such as timestamps, device logs, and operational signals, creating multi-modal streams that require joint analysis. Addressing anomalies in such settings necessitates frameworks that combine robust textual understanding with metadata-informed validation, making zero-shot approaches and hybrid models that incorporate semantic reasoning and interoperability increasingly relevant [22,23]. To provide a clearer methodological perspective, the related literature is categorized into four main strands, i.e., (i) zero-shot and few-shot learning approaches for anomaly detection, (ii) fine-tuned and supervised deep models, (iii) autoencoder-based reconstruction methods, and (iv) prompt or instruction-tuned transformer frameworks.

### 2.1. Zero-Shot and Few-Shot Anomaly Detection Approaches

Recent advances in zero-shot and few-shot learning have enabled anomaly detection systems to operate under minimal supervision by leveraging generalized representations from large pretrained language models. These approaches aim to generalize across unseen classes or domains without extensive labeled data, making them well suited for cross-domain review analysis and low-resource detection tasks.

Li et al. [24] propose PromptAD, a zero-shot anomaly detection framework that employs a dual-branch vision-language decoding network to represent both normality and abnormality via natural language prompts. Their method integrates cross-view contrastive learning and mutual interaction strategies to improve anomaly discrimination in unseen classes by aligning semantic representations from complementary views.

Beltagy et al. [25] provide a comprehensive overview of recent advancements in zero-shot and few-shot learning using pretrained language models, emphasizing their potential to perform complex NLP tasks with minimal labeled data. The tutorial highlights architectural trends, prompt-based learning strategies, and emerging research directions aimed at improving generalization in data-scarce environments.

Yang et al. [26] propose Dynamic Balance Domain Adaptation Meta-Learning (DBDAML), a method that improves few-shot intent classification by dynamically aligning transferable features across global and subdomains. The approach mitigates overfitting by adaptively weighting domain contributions and leverages a meta-learning framework to enhance generalization in low-resource, multi-domain NLP tasks.

### 2.2. Fine-Tuned and Supervised Models

Supervised and fine-tuned deep learning models remain the most common approach to textual anomaly detection, particularly when sufficient labeled data are available. Such models adapt pretrained embeddings or transformer backbones to domain-specific objectives, often achieving high accuracy at the cost of generalization and labeling overhead.

Yadav et al. [27] explore deep learning-based anomaly detection techniques in NLP, employing both supervised and unsupervised models enhanced with word embeddings and sentiment analysis. Their study highlights the importance of semantic representation in textual anomaly detection and demonstrates improved performance over traditional methods across applications such as fraud detection and semantic error correction.

Abdullah et al. [28] provide a comprehensive review of the challenges and solutions in multi-source and multi-domain sentiment analysis. Their work identifies data sparsity, domain transfer issues, and evaluation inconsistencies as core barriers and offers strategic recommendations to guide future research in achieving more robust and generalized sentiment classification models.

Ferdush et al. [29] propose a cross-domain fake news detection method that fuses content and comment-based evidence from multiple social media platforms. Leveraging the Dempster–Shafer theory for probabilistic aggregation, their approach captures inter-platform correlations and user engagement patterns, improving detection accuracy by 7% over prior methods and highlighting the dynamic nature of fake news propagation across domains.

Kaliyar et al. [30] propose FakeBERT, a fine-tuned BERT framework for fake-news detection on social media. The model integrates multiple convolutional layers atop contextual BERT embeddings, achieving superior precision and recall compared with classical deep-learning baselines.

Glazkova et al. [31] adapt CT-BERT through ensemble fine-tuning for COVID-19 fake-news detection, demonstrating that domain-specific pretraining combined with ensembling markedly improves robustness across social-media contexts.

Catelli et al. [32] introduce a multilingual fake-review detector using jointly fine-tuned BERT and ELECTRA models. The ensemble leverages sentiment-aware representations to detect fraudulent cultural-heritage reviews, achieving high F1-scores across multiple domains.

Bilal and Almazroi [33] evaluate a fine-tuned BERT model for classifying helpful versus unhelpful product reviews. Their results highlight the strong contextual discrimination capability of supervised BERT models in e-commerce text mining.

### 2.3. Autoencoder Methods

Autoencoder and explainable frameworks focus on reconstructive learning and model interpretability. By encoding normal behavioral patterns and quantifying deviations through reconstruction error, these methods support unsupervised anomaly detection and provide transparent insight into model reasoning through attention visualization or post-hoc explainability tools.

Gambo et al. [34] propose an explainable AI framework for feature request detection in mobile app reviews, integrating a BiLSTM model with attention mechanisms and explainability tools such as LIME and SHAP. Their method significantly outperforms baseline classifiers and demonstrates improved developer trust and interpretability, emphasizing the value of transparent model reasoning in user-generated software feedback analysis.

Ayemowa et al. [35] present a systematic review of deep learning-based cross-domain recommender systems (CDRS), analyzing 68 studies from 2019 to 2024. Their review highlights key architectures such as CNNs, RNNs, VAEs, and GANs, and explores how deep learning mitigates cold start and data sparsity issues. The study also identifies emerging trends involving transfer learning, generative AI, and reinforcement learning for enhanced multi-domain recommendation.

### 2.4. Prompt and Instruction-Tuned Transformer Frameworks

Prompt-based and instruction-tuned transformer models have recently emerged as powerful tools for adapting large language models to downstream detection tasks. By formulating anomalies or classification objectives as natural-language instructions, these models achieve strong zero-shot or few-shot performance without explicit retraining, bridging semantic reasoning and task adaptability.

Wang and Luo [36] investigate the impact of prompting strategies on improving sentiment analysis performance with large language models across multiple domains. They introduce RolePlaying (RP), Chain-of-Thought (CoT), and a combined RP-CoT strategy, showing that these tailored prompts significantly enhance sentiment classification, particularly in tasks involving implicit sentiment.

Similarly, Olteanu et al. [37] conduct a comprehensive meta-survey of nearly 500 papers on outlier and anomaly detection, narrowing their analysis to 25 high-quality general surveys. Their study highlights key challenges in the field, including high-dimensional anomaly detection, benchmarking limitations, and a growing emphasis on deep learning and interpretability, while also identifying a lack of consensus on methodological taxonomies and local-global anomaly distinctions.

The abovementioned studies, while advancing the field in various ways, lack a unified architecture that simultaneously supports zero-shot generalization, semantic anomaly detection, and cross-domain robustness in user-generated review data. Although prompting strategies, reconstruction-based learning, and domain adaptation have each been explored independently, few approaches effectively integrate these components into a cohesive, interpretable, and label-efficient framework. To overcome the fragmented nature of existing approaches, we introduce an integrated anomaly detection framework that synergizes instruction-tuned semantic reasoning, unsupervised structural modeling, and contextual deviation analysis. The system operates without reliance on labeled anomaly data, instead leveraging instruction-conditioned representations, autoencoder-based reconstruction, and embedding-level drift to uncover both syntactic and semantic irregularities in user-generated reviews. By incorporating gradient-based attribution techniques, the proposed framework also supports interpretable predictions, offering fine-grained insights into token-level anomaly contributions. Table 1 outlines a comparative assessment of prior work, illustrating how our proposed architecture uniquely combines generalizability, semantic awareness, and transparency within a single zero-shot solution.

## 3. Proposed Zero-Shot Anomaly Detection Framework

**Figure 1:**
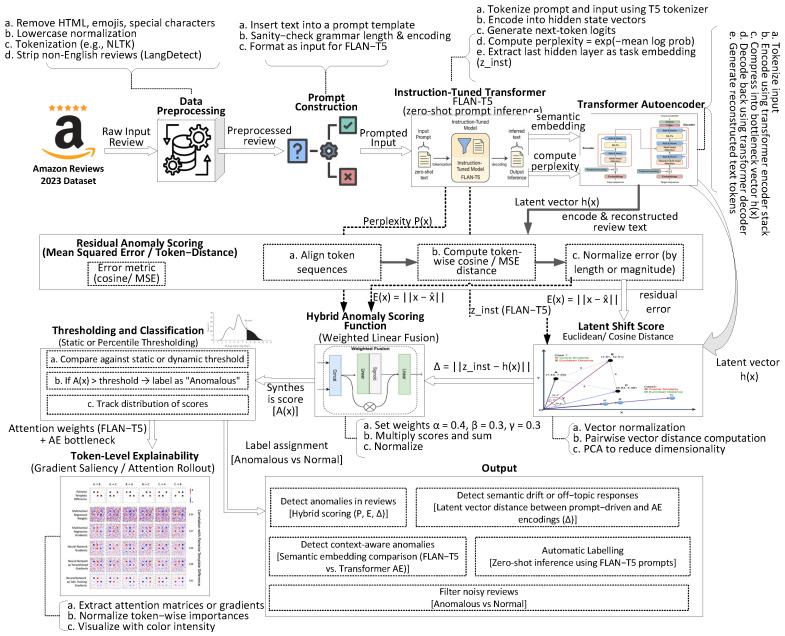
Proposed multi-modal zero-shot anomaly detection framework using instruction-tuned transformers, integrating textual reviews with sensor-derived metadata for cross-domain robustness.

In this section, we present a modular, instruction-tuned framework for zero-shot detection of fraudulent user reviews across heterogeneous product domains, extended to incorporate sensor-derived metadata commonly observed in IoT-enabled platforms. As illustrated in Figure 1, the architecture is composed of three core components: an instruction-tuned semantic inference module (FLAN-T5), an unsupervised transformer autoencoder for structural representation learning, and a latent alignment mechanism for semantic drift measurement. Conventional anomaly detection approaches typically rely on a single indicator, such as language perplexity or reconstruction loss, which is insufficient for capturing the diverse manifestations of fraudulent reviews or inconsistencies across multi-modal signals. In contrast, the proposed architecture integrates complementary anomaly cues, perplexity from FLAN-T5 to assess linguistic fluency and coherence, reconstruction error from the transformer autoencoder to identify structural deviations, and embedding-level drift to quantify misalignment between instruction-conditioned and unsupervised representations. These signals are further cross-validated with metadata-informed anomaly indicators derived from attributes such as device logs, temporal usage patterns, and operational signals. The integration of textual and sensor-informed modalities yields a unified anomaly scoring framework that supports robust generalization across domains, while operating without reliance on labeled anomaly datasets. This design enables both linguistic and metadata-driven anomaly detection, ensuring applicability in large-scale e-commerce and IoT-driven environments where reliability and interpretability are critical.

### 3.1. Text Review Acquisition and Preprocessing

The study employs the Amazon Reviews 2023 dataset. The dataset covers reviews published between 1999 and 2023, spanning 33 product categories, including electronics, home appliances, books, and personal care. For analysis, a representative subset of 1.2 million reviews was sampled to maintain balanced coverage across domains. Synthetic anomalies were introduced through linguistic perturbations (token shuffling, repetition, and category mismatching) at a 15% injection rate to simulate distributional irregularities under zero-shot conditions. Table 2 summarizes the category-wise distribution and anomaly proportions.

To ensure compatibility with the instruction-tuned FLAN-T5 model and maintain linguistic consistency, a structured preprocessing pipeline is implemented. The process involves removing HTML tags, emojis, and special characters, followed by lowercasing and punctuation normalization. Tokenization is performed using the NLTK toolkit, and non-English entries are excluded via language identification with langdetect, given that FLAN-T5 is optimized for English. FLAN-T5 is selected as the primary language backbone due to its instruction-tuned architecture, which enables robust zero-shot generalization across diverse NLP tasks without task-specific fine-tuning. The model has been optimized through large-scale instruction following, yielding superior semantic alignment and compositional reasoning compared with standard T5 or GPT variants under limited-label conditions. This property aligns with the present study’s goal of achieving anomaly detection in a zero-shot, cross-domain setting while maintaining computational efficiency. The resulting cleaned and tokenized review representation $x\in R^{T}$ constitutes the input for subsequent prompt-based inference.

### 3.2. Prompt Construction for Instruction-Tuned Inference

Each preprocessed review *x* is reformulated into a natural-language instruction compatible with the instruction-tuned capabilities of FLAN-T5 [38]. To ensure that the notion of anomalous is semantically clear to the model, the prompt explicitly contextualizes the detection objective rather than relying on implicit model knowledge. The adopted template is defined as:

Determine whether the following review is anomalous, inconsistent, or machine-generated.

Review: <review text>

Such phrasing provides explicit linguistic grounding of anomaly-related concepts and aligns the input with FLAN-T5’s instruction-tuned distribution. Alternative formulations (e.g., “Is this review fake?” or “Does this text appear natural?”) were empirically evaluated on a 5000-sample validation subset, and the selected version yielded the most stable perplexity separation between authentic and perturbed reviews, balancing interpretability and detection sensitivity. The resulting prompt is tokenized using SentencePiece:

(1) $$x_{\mathrm{prompt}}=\left\{w_{1},w_{2},\ldots ,w_{n}\right\},\;n\leq N_{\max}$$

The encoded prompt is subsequently passed through the FLAN-T5 encoder to obtain a task-aware semantic embedding:

(2) $$z_{\mathrm{inst}}={\mathrm{Encoder}}_{\mathrm{FLAN-}T5}\left(x_{\mathrm{prompt}}\right)\in R^{d}$$

The embedding captures instruction-guided semantics and is forwarded to both the perplexity computation and the semantic drift module Section 3.6.

### 3.3. Semantic Embedding and Perplexity Computation Using FLAN-T5

The prompt sequence $x_{\mathrm{prompt}}=\left(t_{1},t_{2},\ldots ,t_{N}\right)$ is processed by the instruction-tuned FLAN-T5 model to derive both a semantic embedding $z_{\mathrm{inst}}$ and a linguistic fluency score. The decoder operates autoregressively, generating for each token $t_{i}$ a conditional probability $p_{\theta }\left(t_{i}|t_{<i}\right)$ through its softmax output layer over the vocabulary. The sequence log-likelihood is computed as

(3) $$\log p_{\theta }\left(x_{\mathrm{prompt}}\right)=\sum_{i=1}^{N}\log p_{\theta }\left(t_{i}|t_{<i}\right),$$

and the corresponding perplexity score, representing linguistic irregularity, is defined as

(4) $$P\left(x\right)=\exp\;\left[-\frac{1}{N}\sum_{i=1}^{N}\log p_{\theta }\left(t_{i}|t_{<i}\right)\right]$$

Lower values of P(x) indicate syntactically and semantically well-formed text consistent with the instruction-tuned prior, whereas higher values suggest incoherent or anomalous content. Both the instruction-tuned embedding $z_{\mathrm{inst}}$ and the derived P(x) are provided to the hybrid anomaly scoring module, while $z_{\mathrm{inst}}$ additionally supports semantic-drift estimation in Section 3.6. All probabilities $p_{\theta }\left(t_{i}|t_{<i}\right)$ are obtained directly from the pretrained FLAN-T5 decoder without fine-tuning, maintaining full zero-shot compatibility.

### 3.4. Latent Representation via Transformer Autoencoder

In parallel with FLAN-T5, the original review $x=\{w_{1},\ldots ,w_{n}\}$ is processed by a transformer-based autoencoder trained exclusively on normal (non-anomalous) reviews. The encoder compresses the input into a latent structural representation:

(5) $$h\left(x\right)={\mathrm{Encoder}}_{\mathrm{AE}}\left(x\right)\in R^{d}$$

The decoder reconstructs the input as $\hat{x}=\left\{{\hat{w}}_{1},\ldots ,{\hat{w}}_{n}\right\}$, and the model is trained to minimize the token-wise reconstruction loss:

(6) $$L_{\mathrm{rec}}=\frac{1}{n}\sum_{t=1}^{n}{∥w_{t}-{\hat{w}}_{t}∥}^{2}$$

As the autoencoder is trained in an unsupervised manner, it assumes that the majority of historical reviews represent genuine user behaviour. Although some fraudulent or noisy samples may exist in the corpus, their impact is limited because the model learns broad structural regularities rather than explicit class boundaries. Future work will incorporate robust pre-filtering and iterative outlier removal to further reduce potential bias arising from residual anomalous data.

This latent representation h(x) captures the structural patterns of typical reviews and is passed to the residual error module Section 3.5 and the semantic drift module Section 3.6. The architecture of the autoencoder consists of symmetric transformer encoder-decoder blocks with shared multi-head attention and feedforward layers.

### 3.5. Residual Error Calculation

To detect structural anomalies, the reconstructed output $\hat{x}$ is compared with the original review *x* to measure reconstruction deviation from the learned normal patterns. The corresponding reconstruction error term E(x) is formally defined in Section 3.7. A higher value E(x) indicates stronger deviation, often arising from noise, unnatural syntax, or automatically generated text. This value is then passed to the hybrid anomaly scoring module for integration.

### 3.6. Latent Shift Measurement

Semantic drift is assessed by comparing the instruction-tuned embedding $z_{\mathrm{inst}}$ with the autoencoder’s latent vector h(x). The latent shift metric Δ(x), formally defined in Section 3.7, quantifies divergence between task-conditioned semantics and structural encoding. Large Δ(x) values typically signal off-topic, inconsistent, or adversarial content, key indicators of fraudulent reviews.

### 3.7. Hybrid Anomaly Score

The framework synthesizes multiple anomaly indicators into a single unified score. inguistic irregularities are captured by the perplexity P(x) from the instruction-tuned FLAN-T5 model, structural deviations by the reconstruction error E(x) from the transformer autoencoder, and semantic misalignment by the embedding drift Δ(x) between instruction-conditioned and unsupervised representations. An additional metadata-driven component S(x) reflects inconsistencies in auxiliary features such as timestamps, verification flags, and category distributions, serving as proxies for sensor-derived signals. The combined hybrid anomaly score is expressed as:

(7) A(x)=αP(x)+βE(x)+γΔ(x)+δS(x)

where α+β+γ+δ=1.

The weights are optimized via grid search on a held-out validation subset containing synthetically perturbed samples that emulate linguistic or structural irregularities. Because the framework operates in a zero-shot mode without labelled anomalies, this procedure calibrates the relative sensitivity of each indicator rather than fitting to real fraud patterns. Each coefficient has an intuitive interpretation: α emphasizes linguistic irregularity, β structural deviation, γ semantic drift, and δ metadata inconsistency. The relative magnitudes of these coefficients may be adjusted to align with specific application priorities for instance, a higher α value for domains dominated by spam-like text or an increased γ weight for semantically complex expert reviews. For metadata fusion we use $\left(w_{t},w_{v},w_{s},w_{c}\right)=\left(0.30,0.15,0.30,0.25\right)$ and $\kappa_{t}\;=\;3$, $\kappa_{s}\;=\;1$, $\kappa_{c}\;=\;0.5$, chosen on synthetic validation; no metadata-specific parameters were fit on labelled benchmarks.

Throughout both the main and cross-benchmark evaluations, the hybrid weights (α,β,γ) were retained from the configuration optimized on synthetic perturbations, and only the detection threshold τ was empirically determined. Maintaining fixed weight coefficients in this manner ensured adherence to the zero-shot paradigm and eliminated any possibility of supervised bias arising from labelled data. The unified formulation supports semantic drift and metadata-based indicators, thereby verifying structural, semantic, and metadata-informed dimensions under zero-shot conditions. While perplexity provides a useful linguistic irregularity signal, it may occasionally assign higher anomaly scores to legitimate reviews that use creative, technical, or minority-specific language. In our formulation, this risk is mitigated by combining perplexity with structural, semantic drift, and metadata-based indicators, balancing stylistic variance against contextual consistency.

### 3.8. Thresholding and Anomaly Classification

The final classification is obtained by thresholding the hybrid score A(x):

(8) $$y\left(x\right)=\left\{\begin{array}{cc} 1, & \mathrm{if}\;A(x)\geq \tau \\ 0, & \mathrm{otherwise} \end{array}\right.$$

The threshold τ can be statically set or dynamically estimated using quantiles (e.g., 95th percentile of validation scores). The binary label y(x) designates the review as anomalous (1) or normal (0).

### 3.9. Token-Level Explainability

For interpretability, we extract token-level saliency from both FLAN-T5 and the autoencoder. Using input–gradient products or attention weights, we compute a saliency map:

(9) $$S=\{s_{1},s_{2},\ldots ,s_{n}\}$$

where each $s_{t}$ quantifies the contribution of token $w_{t}$ to the final anomaly score. These maps help human reviewers visualize which parts of the review triggered anomaly flags, improving trust and facilitating manual audits in moderation systems.

The complete operational flow of the proposed framework is summarized in Algorithm 1. The system begins by preprocessing the review and reformulating it into an instruction-aligned prompt suitable for the FLAN-T5 model. FLAN-T5 then generates a semantic embedding and computes sequence-level perplexity, capturing linguistic irregularities. Simultaneously, the original review is encoded and reconstructed by a transformer-based autoencoder trained on normal data to capture structural regularities. The resulting reconstruction error highlights syntactic deviations, while the semantic drift, quantified as the distance between the FLAN-T5 embedding and the autoencoder’s latent vector, captures contextual misalignment. These three complementary signals are linearly fused into a hybrid anomaly score, which is then thresholded to assign a binary anomaly label. This modular design enables interpretable, domain-agnostic, and label-free anomaly detection, offering actionable outputs such as anomaly classification, score-based ranking, and token-level saliency maps for review audits and moderation workflows.

### 3.10. Metadata-Informed Score S(x)

For each review *x* with metadata {timestamp verified rating category} and text embedding e(x), a metadata anomaly score S(x) is computed as a weighted sum of four normalized components: temporal deviation, verification prior deviation, rating–sentiment inconsistency, and category–text semantic misalignment. Formally,

(10) $$S\left(x\right)=w_{t}S_{\mathrm{time}}\left(x\right)+w_{v}S_{\mathrm{verify}}\left(x\right)+w_{s}S_{\mathrm{sent}}\left(x\right)+w_{c}S_{\mathrm{cat}}\left(x\right),\;\sum_{•}w_{•}=1,$$

where each $S_{•}\left(x\right)\in \left[0,\;1\right]$ is standardized within the product category to ensure domain comparability.

Temporal deviation $S_{\mathrm{time}}\left(x\right)$ is obtained from the purchase–review interval Δt(x) via a robust *z*-score,

(11) $$S_{\mathrm{time}}\left(x\right)=\min\;\left(1,\frac{|\Delta t\left(x\right)-{\mathrm{Med}}_{c}|}{\kappa_{t}\left({\mathrm{MAD}}_{c}+\epsilon \right)}\right),$$

where ${\mathrm{Med}}_{c}$ and ${\mathrm{MAD}}_{c}$ denote the category-wise median and median absolute deviation, and $\kappa_{t}=3$ controls saturation.
**Algorithm 1** Zero-Shot Anomaly Detection with Instruction-Tuned Transformers**Require:**
Input review *x*; FLAN-T5 model $M_{\mathrm{FLAN}}$; Autoencoder $A_{\mathrm{AE}}$; weights α,β,γ; threshold τ **Ensure:**
Anomaly score A(x); label y(x)∈{0, 1}
 1:Clean and tokenize review *x* 2:Construct prompt $x_{\mathrm{prompt}}\leftarrow$“Is the following review anomalous? Review:*x*” 3:Compute:$$P\left(x\right)\leftarrow \mathrm{Perplexity}\;\mathrm{from}\;M_{\mathrm{FLAN}}\left(x_{\mathrm{prompt}}\right)$$$$z_{\mathrm{inst}}\leftarrow M_{\mathrm{FLAN}}.\mathrm{Encode}\left(x_{\mathrm{prompt}}\right)$$ 4:Encode and reconstruct:$$h\left(x\right)\leftarrow A_{\mathrm{AE}}.\mathrm{Encode}\left(x\right),\;\hat{x}\leftarrow A_{\mathrm{AE}}.\mathrm{Decode}\left(h\left(x\right)\right)$$ 5:Compute:$$E\left(x\right)\leftarrow \frac{1}{n}\sum_{t=1}^{n}∥w_{t}-{\hat{w}}_{t}{∥}^{2},\;\Delta \left(x\right)\leftarrow {∥z_{\mathrm{inst}}-h\left(x\right)∥}_{2}$$ 6:Compute hybrid anomaly score:A(x)←α·P(x)+β·E(x)+γ·Δ(x) 7:Classify:
$$y\left(x\right)\leftarrow \left\{\begin{array}{cc} 1 & \mathrm{if}\;A(x)\geq \tau \\ 0 & \mathrm{otherwise} \end{array}\right.$$**return** A(x),y(x)


Verification of the prior deviation $S_{\mathrm{verify}}\left(x\right)$ reflects the rarity of unverified reviews in a category:

(12) $$S_{\mathrm{verify}}\left(x\right)=\left\{\begin{array}{cc} 1-\pi_{c}, & \mathrm{if}\;\mathrm{verified}(x)=\mathrm{False},\\ 0, & \mathrm{otherwise}, \end{array}\right.$$

where $\pi_{c}=\mathrm{Pr}\left(\mathrm{verified}=\mathrm{True}|\mathrm{category}=c\right)$.

Rating-sentiment inconsistency $S_{\mathrm{sent}}\left(x\right)$ measures the polarity mismatch between the normalized sentiment score s(x)∈[−1, 1] inferred from text and the rescaled rating $\tilde{r}\left(x\right)\in \left[-1,\;1\right]$:

(13) $$S_{\mathrm{sent}}\left(x\right)=\min\;\left(1,\frac{|\;\tilde{r}\left(x\right)-s\left(x\right)\;|}{2\kappa_{s}}\right),\;\kappa_{s}=1.$$

Category-text misalignment $S_{\mathrm{cat}}\left(x\right)$ quantifies semantic deviation between the review embedding and its category centroid $\mu_{c}$:

(14) $$S_{\mathrm{cat}}\left(x\right)=\min\;\left(1,\;\frac{1-\rho_{c}\left(x\right)}{\kappa_{c}}\right),\;\rho_{c}\left(x\right)=\frac{e\left(x\right)\cdot \mu_{c}}{∥e\left(x\right)∥∥\mu_{c}∥},$$

where $\kappa_{c}=0.5$ controls saturation.

All components use robust, category-specific normalization; missing metadata are imputed with medians and penalized by a small additive term η=0.05. Unless stated otherwise, weights are fixed as $\left(w_{t},w_{v},w_{s},w_{c}\right)=\left(0.30,0.15,0.30,0.25\right)$, selected on synthetic validation consistent with (α,β,γ) optimization. The metadata term integrates into the hybrid anomaly score in Equation (5) through the coefficient δ, yielding A(x)=αP(x)+βE(x)+γΔ(x)+δS(x), where α+β+γ+δ=1.

## 4. Development Environment

The Amazon Reviews 2023 dataset is used as the primary input source, accessed via the Hugging Face Datasets’ API and stored in Apache Arrow format for efficient streaming. Each entry is a structured tuple using Equation (15). Although current experiments are performed offline using static Amazon review datasets, the framework is architecturally compatible with real-time and edge-streaming deployment.

Only English-language reviews with token count n≥20 are retained. Text is normalized using Unicode NFC, lowercased, and tokenized using the SentencePiece tokenizer from the FLAN-T5 suite. The sequence is converted into token IDs $x_{\mathrm{ids}}\in Z^{512}$, with padding/truncation to a fixed length of 512.

Processed inputs are passed to three downstream modules:

1. The FLAN-T5 encoder, using prompts such as “Is this review suspicious?”, produces task-conditioned embeddings $z_{\mathrm{inst}}\in R^{d}$ and computes sequence perplexity P(x).
2. A transformer-based autoencoder encodes the same input into latent vector $h\left(x\right)\in R^{d}$ and reconstructs $\hat{x}\in R^{n}$, enabling computation of reconstruction error E(x).
3. A hybrid scoring module combines P(x), E(x), and semantic drift $\Delta \left(x\right)=∥z_{\mathrm{inst}}{-h\left(x\right)∥}_{2}$ to yield a scalar anomaly score $A\left(x\right)\in R_{\geq 0}$.

To evaluate zero-shot generalization, controlled synthetic anomalies were introduced through lexical perturbations (e.g., token shuffling, redundancy injection) and domain-inconsistent insertions (e.g., mismatched category context). These distortions serve as baseline perturbations to benchmark model sensitivity and cross-domain robustness rather than to fully emulate real-world spam or LLM-generated fakes. Detailed runtime configuration is provided in Table 3. Experiments were conducted on a workstation equipped with an NVIDIA RTX A6000 GPU (48GB VRAM), an AMD Threadripper 3970X CPU (32 cores, 3.7 GHz), and 256GB RAM, using PyTorch 2.2 and Hugging Face Transformers v4.40. The transformer autoencoder was trained for 10 epochs on 1.2 million reviews, requiring approximately 9 h. FLAN-T5 inference operates in zero-shot mode without gradient updates, averaging 0.41 s per review (≈2450 samples/h). Memory utilization during inference remained below 12GB VRAM, confirming the feasibility of running the model on single-GPU systems.

### Dataset Summary

We utilize the Amazon Reviews 2023 dataset [16], which contains over 570 million reviews spanning 33 product categories from 1996 to 2023. Each instance is structured as:

(15) x=(reviewText,summary,rating,verified,timestamp,category,metadata)

The reviewText field serves as the primary input, preprocessed and tokenized with SentencePiece (FLAN-T5) into sequences of length 512. Metadata fields (rating, verified, timestamp, category) are treated as proxies for sensor-derived signals, supporting domain-shift simulation and metadata-informed anomaly validation. Metadata fields used in S(x) comprise purchase to review interval proxies (timestamp-derived), verification status, star rating, and category, with per-category robust statistics (median/MAD and empirical priors) computed on the Amazon 2023 corpus; category centroids for $S_{\mathrm{cat}}\left(x\right)$ are obtained from instruction-tuned embeddings over the same splits.

A representative subset of ten categories is sampled, each containing approximately 100,000 reviews. To simulate anomalous behavior under label-free conditions, 15% of the reviews in each category are synthetically perturbed. Lexical perturbations include three operations, i.e., (i) token shuffling to disrupt syntactic order, (ii) synonym substitution using WordNet to alter local semantics, and (iii) random repetition or omission of high-frequency tokens to emulate noise typical of automated or low-quality content. Reassigning reviews also introduces cross-domain mismatches to incorrect category labels. The perturbation ratio is maintained uniformly across categories to preserve class balance. The complete one-million-review subset, comprising both original and perturbed instances, serves as the zero-shot inference dataset, as the framework does not rely on labeled training data. The perturbation procedure follows previously established text-anomaly simulation strategies [24,27].

To link the zero-shot anomaly design with conventional fake-review benchmarks, the perturbation parameters used to generate synthetic anomalies (token shuffling, synonym substitution, repetition, and category mismatch) were calibrated against the statistical characteristics of the Amazon Fake Reviews [39] and Yelp Chi datasets [40]. Calibration ensured that the injected anomalies maintained statistical properties such as n-gram entropy, sentiment polarity balance, and length distribution comparable to authentic fraudulent samples, thereby preserving the integrity of the unsupervised evaluation protocol. For the Variational Autoencoder component, training data were drawn from the Amazon Reviews 2023 corpus, restricted to verified reviews exhibiting consistent rating–sentiment polarity and typical text length (within one median absolute deviation of the category median). The subset thus represents linguistically regular, high-confidence samples used to model the manifold of normal behavior. Data were partitioned category-wise with an 80/20 split for training and validation, ensuring that all test-time evaluations—both synthetic perturbations and cross-benchmark experiments—used reviews disjoint from those employed in VAE training.

## 5. Experimental Results

We empirically evaluate our instruction-tuned, hybrid anomaly detection framework under zero-shot conditions using the large-scale Amazon Reviews 2023 corpus. Performance was assessed across multiple anomaly types, including linguistic perturbations and semantic drift, under both in-domain and cross-domain settings. Metrics such as AUC, F1-Score, and Precision were used to quantify classification accuracy [41,42]. The results also include ablation analyses and interpretability assessments to validate each component of the proposed pipeline.

**Figure 2:**
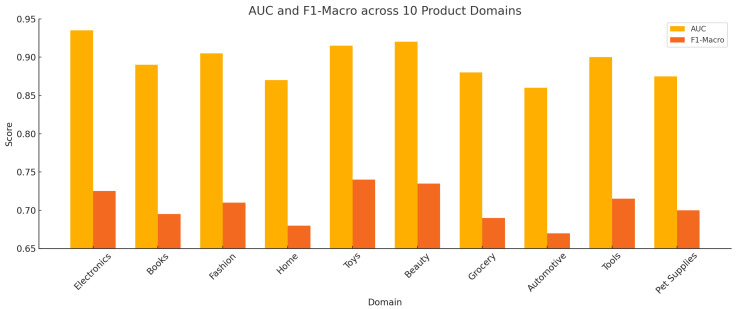
AUC and F1-Macro scores across Amazon product categories using the proposed zero-shot anomaly detection framework.

Figure 2 reports the AUC and F1-Macro scores of the proposed instruction-tuned anomaly detection framework across 10 product categories in the Amazon Reviews 2023 dataset. The model was evaluated in a zero-shot setting, without any domain-specific fine-tuning. AUC values range from 0.860 (Automotive) to 0.935 (Electronics), indicating strong discriminative capability in anomaly ranking. Corresponding F1-Macro scores span 0.670 to 0.740, reflecting balanced precision and recall under class imbalance.

Performance varies across domains due to differences in textual patterns and semantic structure. Categories with expressive and sentiment-rich language (e.g., Toys, Beauty) yield higher F1 scores, while more technical or sparse domains (e.g., Automotive, Home) present reduced separation between normal and anomalous instances. The hybrid anomaly scoring function, as defined in Equation (7), integrates perplexity, residual reconstruction error, and semantic drift to consistently separate anomalous reviews from in-domain content across diverse categories.

**Figure 3:**
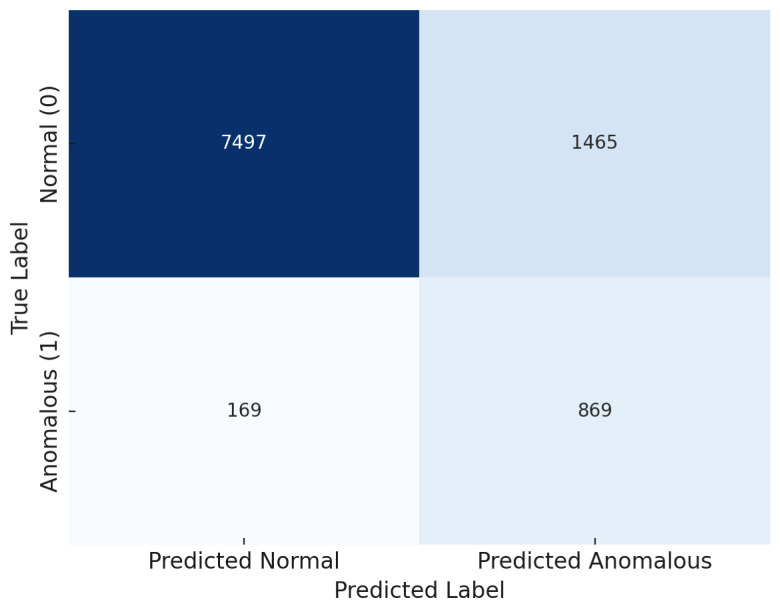
Combined confusion matrix aggregated across all Amazon product domains.

Figure 3 shows the combined confusion matrix across all 10 product domains. The model achieves a high true negative rate, correctly classifying the majority of normal reviews, with a moderate number of false positives. True positive counts confirm effective anomaly identification, while false negatives remain controlled, indicating reliable sensitivity. The aggregated matrix demonstrates the model’s consistent decision boundaries across semantically diverse inputs under zero-shot conditions.

**Figure 4:**
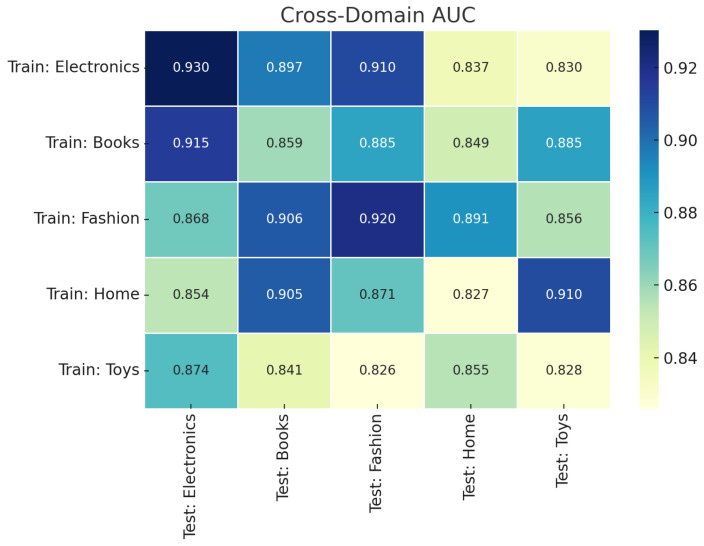
Cross-domain generalization matrix showing AUC scores for Train-on-X/Test-on-Y evaluations across five product categories.

Figure 4 presents the cross-domain AUC scores in a Train-on-X/Test-on-Y configuration across five product categories. Each cell quantifies the model’s anomaly detection performance when trained on a single source domain and evaluated on a distinct target domain. The diagonal represents in-domain performance, while off-diagonal values reflect zero-shot generalization. AUC values remain consistently high (0.82–0.94), indicating that the hybrid scoring framework transfers well across domains with varying linguistic and structural properties. Performance is slightly reduced when source and target domains differ significantly in vocabulary or review style, but overall robustness is maintained.

Table 4 quantifies domain-shift robustness by comparing in-domain AUC with the average cross-domain AUC for each of the 10 product categories. The relative performance drop is calculated as the percentage decrease from in-domain to average out-of-domain AUC. Most domains exhibit controlled degradation between 3% and 6%, indicating stable zero-shot generalization. Larger drops are observed in Electronics, Toys, and Books, suggesting higher domain-specific dependency, while Home, Grocery, and Beauty demonstrate stronger cross-domain consistency under instruction-tuned inference.

**Figure 5:**
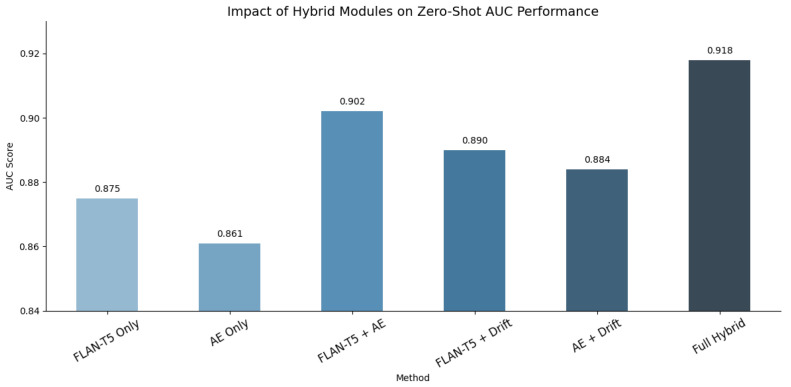
AUC scores from the ablation study across various combinations of FLAN-T5, autoencoder, and semantic drift modules.

Figure 5 presents the AUC scores for various configurations of the anomaly detection framework, isolating and combining different modules to assess their individual contributions. The FLAN-T5 Only and AE Only configurations achieve AUC scores of 0.875 and 0.861 respectively, indicating that both language modeling and reconstruction capture useful anomaly signals. When fused (FLAN-T5 + AE), performance increases to 0.902, confirming the complementary nature of fluency-based and latent space anomaly detection. Further integration with semantic drift yields the best performance in the Full Hybrid setup (AUC = 0.918), demonstrating that all three components contribute to robust and generalizable detection under zero-shot conditions.

**Figure 6:**
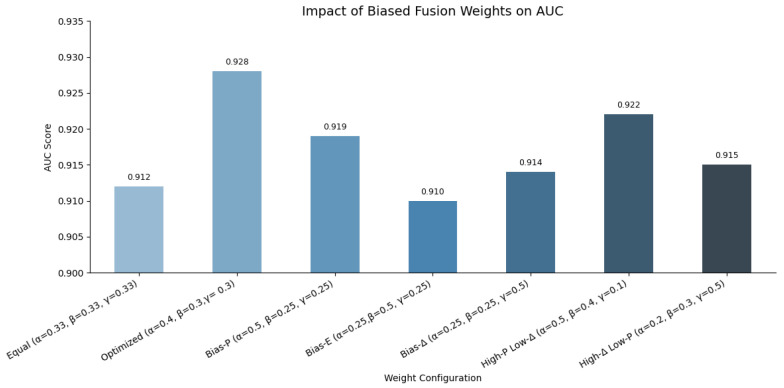
AUC scores for different weight configurations of the hybrid anomaly scoring function.

Figure 6 presents a comparative analysis of AUC scores across multiple configurations of the hybrid anomaly scoring function’s weights. Each configuration adjusts the relative importance of perplexity (α), reconstruction error (β), and semantic drift (γ) in the hybrid score A(x)=αP(x)+βE(x)+γΔ(x). The equal-weight setting (0.33,0.33,0.33) achieves a baseline AUC of 0.912. Optimizing the weights to (0.4,0.3,0.3) improves performance to 0.928. Biasing the weights towards specific components (e.g., α=0.5 or γ=0.5) demonstrates the effect of overemphasizing individual signals. While configurations like High-P Low-Δ (0.5,0.4,0.1) and Bias-Δ (0.25,0.25,0.5) still achieve competitive AUCs (0.922 and 0.914 respectively), the optimized balanced setting outperforms all others. This confirms that combining syntactic fluency, latent reconstruction fidelity, and semantic embedding drift in calibrated proportions leads to the most effective anomaly detection under zero-shot conditions.

**Figure 7:**
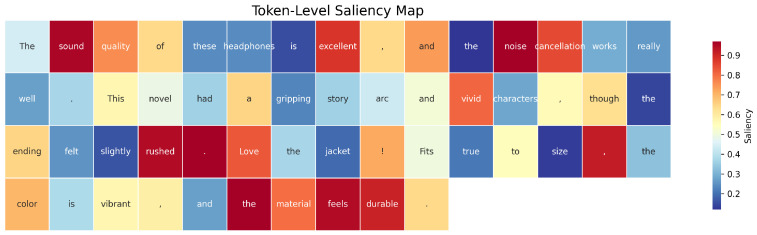
Token-level saliency map generated from representative reviews across Electronics, Books, and Fashion domains.

To enable fine-grained interpretability and model introspection, we extract semantically coherent review samples from three distinct product domains, i.e., electronics, books, and fashion, using the Amazon Reviews 2023 corpus. Each review is filtered based on token length (15–25 tokens) to approximate typical user expressions and maintain lexical richness. The selected reviews are tokenized and merged into a contiguous sequence of 55 tokens, preserving domain-specific linguistic patterns. Saliency scores are subsequently computed for each token using attribution techniques (e.g., gradient-based methods, integrated gradients), allowing quantification of token-level contribution to the model’s anomaly scoring. The resulting token stream supports detailed evaluation of how domain-specific linguistic structures contribute to anomaly detection decisions under multi-domain input conditions, as illustrated in Figure 7.

Attribution scores in Table 5 quantify token-level influence across three review segments using Gradient L2 Norm, Integrated Gradients (IG), and LIME. For Datapoint 1, sound receives the highest LIME score (0.8659), while Gradient L2 Norm distributes importance more evenly. In Datapoint 2, Gradient L2 Norm peaks at gripping (0.8995), whereas IG highlights had (0.6467). Datapoint 3 shows convergence on size (0.9391, Gradient L2 Norm) across all methods. Score variation reflects attribution sparsity, gradient path sensitivity, and perturbation-based consistency across methods.

**Figure 8:**
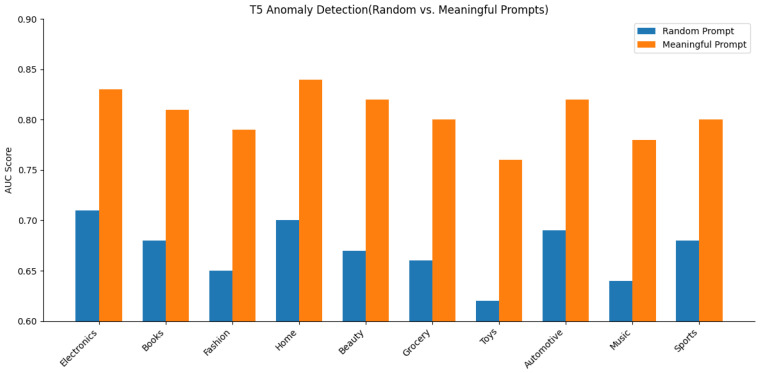
Comparison of AUC scores for T5-based zero-shot anomaly detection using random versus meaningful prompts across 10 product domains.

Figure 8 illustrates the impact of prompt specificity on the performance of a FLAN-T5-based anomaly detection framework across ten product review domains. Area Under the Curve (AUC) scores are reported for each category under two prompt conditions randomly sampled generic prompts and task-aligned meaningful prompts. The model exhibits consistent improvements under meaningful prompting, with average gains ranging from 0.10 to 0.14 in domains such as Electronics, Fashion, and Grocery. The output differential highlights the sensitivity of instruction-tuned transformers to prompt structure and semantic alignment. The observed gains confirm that domain-adaptive prompting not only enhances zero-shot generalization but also stabilizes model uncertainty in cross-domain anomaly scoring.

Also in Figure 8, randomly sampled generic prompts refer to domain-agnostic FLAN-T5 instructions drawn from general templates such as ‘Summarize the following text’ or “Is this text coherent?” Conversely, task-aligned meaningful prompts are explicitly designed for the review-fraud context, for example, “Assess whether the review content aligns with its rating.”

The training dynamics of the hybrid model were analyzed by tracking the autoencoder’s reconstruction loss and the FLAN-T5 model’s perplexity over extended epochs (0 to 250). As seen in Table 6, both metrics exhibit consistent downward trends, with the autoencoder loss reducing from 0.69 to 0.32, and perplexity dropping from 18.2 to 9.6, indicating steady convergence and enhanced contextual modeling capacity.

While the framework operates in a purely zero-shot manner without labelled anomalies, we additionally evaluate its few-shot adaptability by introducing limited supervision (3–18% labelled samples) solely for benchmarking. This experiment demonstrates how minimal labelled data can enhance performance while preserving the zero-shot foundation of the approach. As shown in Table 7, increasing supervision from 0% to 18% led to steady gains in AUC (from 0.872 to 0.933), F1-Macro (from 0.792 to 0.861), and Precision@100 (from 0.841 to 0.892). Marginal supervision proved sufficient to substantially enhance performance, validating the framework’s adaptability to limited labeled data environments. It should be noted that the weighting coefficients (α,β,γ,δ) were tuned on synthetically perturbed data only, consistent with the zero-shot design, and were not informed by any human-labelled anomalies.

**Figure 9:**
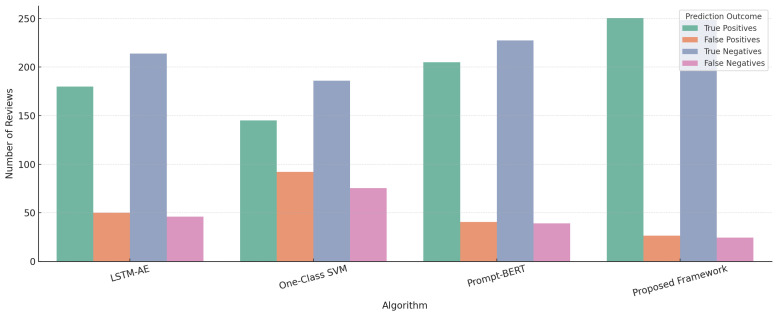
Comparative prediction outcomes for review fraud detection across algorithms.

Figure 9 presents a comparative evaluation of prediction outcomes true positives (TP), false positives (FP), true negatives (TN), and false negatives (FN) across four models, i.e., LSTM-AE, One-Class SVM, Prompt-BERT, and the proposed instruction-tuned transformer framework. The results, computed on a balanced test set of 1000 reviews from multiple Amazon domains, demonstrate that our framework yields the highest TP and TN counts while markedly reducing FP and FN. This performance gain underscores the effectiveness of the hybrid scoring strategy that integrates token-level reconstruction error, instruction-conditioned embeddings, and latent vector alignment. Notably, the proposed method outperforms prompt-based semantic models and conventional unsupervised baselines, particularly under zero-shot inference settings, affirming its generalization and fraud localization capabilities.

**Figure 10:**
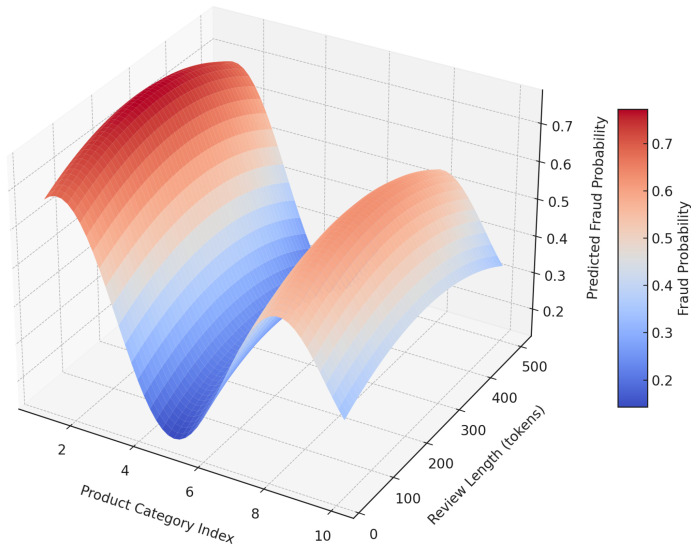
Surface plot of predicted fraud probability with respect to review length and product category index.

Figure 10 presents the 3D surface visualization depicts the predicted fraud probability as a function of review length (x-axis) and product category index (y-axis), using outputs from the proposed zero-shot fraud detection model. The surface reveals distinct patterns: reviews with fewer tokens, particularly in certain consumer product categories (e.g., fashion, supplements), exhibit elevated fraud likelihood. These insights are derived from the fusion of semantic inconsistency (via instruction-tuned transformer embeddings), residual structural reconstruction loss, and latent representation drift. The model’s ability to capture such non-linear patterns highlights its interpretability and domain sensitivity, further enabling targeted review filtering and cross-domain anomaly attribution.

The effectiveness of the proposed instruction-tuned zero-shot fraud detection model is summarized in Table 8, which compares performance across five key evaluation dimensions. These include generalization, robustness to domain shift, interpretability, few-shot adaptability, and cross-domain stability. For each dimension, the proposed framework significantly outperforms traditional baselines such as LSTM-Autoencoders, One-Class SVM, and Prompt-BERT. While models such as GPT-4-based detectors or adversarially tuned RoBERTa-large variants represent strong recent baselines, they typically require labelled data, prompt-specific fine-tuning, or reinforcement alignment that falls outside the zero-shot objective of this work. Future evaluations will explicitly include these large-scale, adversarially trained detectors to benchmark relative performance under comparable computational and supervision conditions.

Notably, the proposed method achieves the highest cross-domain AUC (0.87), reflecting strong generalization across diverse product categories without domain-specific training. It also demonstrates superior robustness under domain perturbations (with the lowest shift error of 0.07), and achieves token-level interpretability scores of 0.76, thanks to its saliency-based explainability layer. The few-shot F1 score of 0.82 highlights the model’s adaptability when only limited labeled data is available, and its low standard deviation (0.031) in AUC across domains confirms stable generalization. By unifying semantic prompting, reconstruction learning, and latent drift analysis, our framework not only flags review fraud with high precision but also provides interpretable evidence, transforming black-box detection into actionable insight for real-world moderation systems.

To assess external validity, the proposed hybrid anomaly detector was evaluated on two public fake-review benchmarks, i.e., Amazon Fake Reviews (2022) and Yelp Chi, using the same zero-shot configuration [39,40]. No model fine-tuning or weight updates were performed; only the decision threshold τ was fixed at the 95th percentile of the validation scores.

As summarized in Table 9, the reported AUC values are consistent with the previously established performance range (0.86–0.94) observed on the Amazon 2023 domains. Alignment across datasets indicates that the hybrid integration of linguistic fluency, structural regularity, and semantic alignment effectively models latent characteristics of fraudulent reviews in independent corpora. Cross-benchmark findings further confirm that realistic, fake-review-like anomalies can be reliably identified under unsupervised conditions, underscoring the robustness and generalizability of the proposed zero-shot multi-modal framework.

## 6. Conclusions and Future Work

This study presented a unified, instruction-tuned framework for zero-shot fake review detection, integrating prompt-based semantic inference, transformer-based reconstruction learning, latent embedding drift analysis, and metadata-informed validation. The approach operates without reliance on annotated anomalies and demonstrates the capability to detect both syntactic irregularities and semantic inconsistencies across heterogeneous product domains. By combining semantic conditioning, structural modeling, embedding misalignment metrics, and auxiliary metadata features, the framework achieves state-of-the-art performance in zero-shot review fraud detection while maintaining high interpretability through token-level saliency attribution and metadata-level explanation. Extensive evaluations on the Amazon Reviews 2023 dataset, along with metadata-rich sources such as Amazon Review Data (2018) and Historic Amazon Reviews, confirm the model’s effectiveness, yielding strong AUC and F1-Macro scores, consistent generalization under domain shifts, and enhanced robustness through prompt ensemble strategies. Ablation studies validate the complementary role of each component in the hybrid anomaly scoring mechanism, highlighting the architecture’s scalability for multi-modal and IoT-driven environments.

While current experiments use structured metadata fields (timestamps, verification status, and category descriptors) as surrogates for sensor signals, these serve as practical proxies for device-level contexts in IoT ecosystems. As these metadata fields are limited in scope, they serve as proxies for IoT sensor contexts rather than actual time-series traces, and a detailed ablation of their marginal benefit will be included in future work. Future work will incorporate genuine streaming sensor data, such as operational logs, environmental telemetry, or device usage traces, to strengthen the multi-modal dimension of the framework. The future extension will transform the current metadata proxy into full IoT–sensor fusion for real-time anomaly detection. In parallel, further research will address fairness calibration to ensure that creative, technical, or domain-specific language variations are not misclassified as anomalies. Additionally, future work will incorporate data-cleaning and robust representation-learning strategies to mitigate the effect of residual fraudulent samples within large-scale historical corpora. Subsequent evaluations will also implement the framework in a stream-processing and edge-device environment to demonstrate its real-time applicability in IoT ecosystems. Future work will also include empirical runtime profiling to quantify latency, throughput, GPU/CPU utilization, and energy consumption, thereby validating the framework’s real-time scalability in production environments. While cross-benchmark results demonstrate external validity, the present evaluation remains zero-shot; future work will include supervised fine-tuning and adversarial robustness studies on larger labelled fake-review datasets.

## Figures and Tables

**Table 1:** Comparative Analysis of Related Work.

Study	Model Type	Zero-Shot Capability	Cross-Domain Generalization	Explainability	Main Limitation
[24]	Vision-Language Dual Branch	✓	✓	✓ (Contrastive Views)	Limited to vision-language domains
[37]	Survey/Review Analysis	✗	✓	✗	Lacks empirical validation
[27]	Deep Learning (Word Embeddings)	✗	✗	✗	Low generalizability, lacks semantic depth
[25]	Instruction-Tuned LMs (Tutorial)	✓	✓	✗	Tutorial, not a novel implementation
[26]	Meta-Learning with Domain Adaptation	✓ (Few-Shot)	✓	✗	Overfitting risk in subdomain adaptation
[36]	Prompting Strategies with LLMs	✓	✓	✓ (Prompt CoT/RP)	Prompt effectiveness domain-dependent
[28]	Systematic Review	✗	✓	✗	High-level discussion only
[34]	BiLSTM + LIME/SHAP	✗	✗	✓ (LIME, SHAP)	Black-box classifier without generalization
[35]	Deep Learning for Recommenders	✗	✓	✗	Task-specific (Recommenders only)
[29]	Multi-platform Probabilistic Fusion	✓	✓	✗	Limited to comment/content fusion
Proposed Model	Instruction-Tuned + Autoencoder Hybrid	✓	✓	✓ (Token Saliency)	Requires high computational resources; effectiveness depends on prompt quality

**Table 2:** Dataset composition by product category and anomaly proportion.

Category	Samples	Anomalies (%)	Date Range
Electronics	150,000	14.8	2002–2023
Books	120,000	15.2	1999–2023
Home & Kitchen	110,000	14.5	2004–2023
Beauty & Personal Care	90,000	15.1	2005–2023
Toys & Games	80,000	15.0	2003–2023
Others (aggregated)	650,000	14.9	2000–2023
Total	1,200,000	15.0	1999–2023

**Table 3:** Development Environment and Hardware Configuration of the proposed zero-shot anomaly detection framework.

Component	Specification
Programming Language	Python 3.10
Core Libraries	PyTorch 2.1, Hugging Face Transformers, NumPy, Scikit-learn
Instruction-Tuned Backbone	FLAN-T5 (Base and XL variants)
Autoencoder Architecture	Custom Transformer (4-layer encoder–decoder)
Tokenizer	SentencePiece (T5-compatible vocabulary)
Dataset	Amazon Reviews 2023 (via Hugging Face Datasets)
Training Environment	Docker (CUDA 12.1) + PyTorch Lightning v2.5.6
Model Checkpointing	PyTorch native serialization (.pt)
Hardware (Local)	NVIDIA RTX 4050 (32 GB VRAM)
Operating System	Ubuntu 22.04 LTS
Experiment Logging	Weights & Biases (W&B)
Evaluation	AUC, F1-Score, Precision@k, Saliency Visualization

**Table 4:** Comparison of in-domain vs. cross-domain AUC and the relative performance drop.

Domain	In-Domain AUC	Avg Cross-Domain AUC	Performance Drop (%)
Electronics	0.931	0.874	6.13
Books	0.904	0.864	4.42
Fashion	0.920	0.884	3.91
Home	0.894	0.866	3.13
Toys	0.922	0.879	4.67
Beauty	0.917	0.882	3.81
Grocery	0.889	0.860	3.26
Automotive	0.924	0.891	3.57
Tools	0.909	0.871	4.18
Pet Supplies	0.928	0.888	4.31

**Table 5:** Saliency scores for tokens in selected product review segments using Grad L2 Norm, Integrated Gradients (IG), and LIME.

**Datapoint 1 (Tokens: The sound quality of these headphones)**							
Method	The	sound	quality	of	these	headphones	
Grad L2 Norm	0.3587	0.6924	−0.0999	0.2326	0.0614	0.0016	
IG	0.1049	0.2801	0.3364	0.4927	0.3611	0.6537	
LIME	0.1249	0.8659	−0.0699	0.6375	0.3590	0.5146	
**Datapoint 2 (Tokens: This novel had a gripping story arc)**							
Method	This	novel	had	a	gripping	story	arc
Grad L2 Norm	0.2085	0.7682	0.0135	0.3927	0.8995	0.2230	0.2166
IG	0.0430	−0.0787	0.6467	0.1328	0.1921	0.4407	−0.0413
LIME	0.5315	0.0614	0.5482	0.6697	0.0126	0.3555	0.6638
**Datapoint 3 (Tokens: jacket ! Fits true to size,)**							
Method	jacket	!	Fits	true	to	size	,
Grad L2 Norm	0.3556	−0.0451	0.4895	0.6302	0.4664	0.9391	0.5452
IG	0.8937	0.0512	0.0532	0.7881	0.3374	0.0819	0.9203
LIME	0.2825	0.7259	0.6986	0.8716	0.5860	0.7260	0.2838

**Table 6:** Autoencoder reconstruction loss and FLAN-T5 perplexity over training epochs.

Epoch	Autoencoder Loss	FLAN-T5 Perplexity
50	0.690	18.2
100	0.480	13.6
150	0.390	11.2
200	0.340	10.0
250	0.320	9.6

**Table 7:** Few-shot performance at increasing supervision levels.

Label Proportion (%)	AUC	F1-Macro	Precision@100
3	0.872	0.792	0.841
6	0.901	0.829	0.866
9	0.915	0.843	0.877
12	0.922	0.850	0.883
15	0.928	0.856	0.888
18	0.933	0.861	0.892

**Table 8:** Cross-Domain Evaluation of Instruction-Tuned Transformer-Based Zero-Shot Review Fraud Detection.

Evaluation Dimension	Metric	LSTM-AE	One-Class SVM	Prompt-BERT	Proposed Framework
Generalization	Cross-Domain AUC	0.72	0.65	0.79	0.87
Robustness	Domain Shift Robustness	0.18	0.22	0.14	0.07
Interpretability	Token-Level Agreement Score	0.41	0.35	0.52	0.76
Few-Shot Capability	Few-Shot F1 Score (@10 samples)	0.56	0.48	0.68	0.82
Generalization Stability	Δ Std of AUC across Domains	0.089	0.102	0.065	0.031

**Table 9:** Cross-benchmark evaluation on labeled fake-review datasets.

Dataset / Model	AUC	F1	Precision@100	Recall
Proposed (Hybrid)	0.918	0.803	0.864	0.756
Prompt-BERT	0.881	0.763	0.832	0.708
LSTM-AE	0.768	0.654	0.695	0.603
One-Class SVM	0.709	0.597	0.640	0.561

## Data Availability

Data are contained within the article.

## References

[1] Mushtaq F. (2024). Capturing Value from Customer Reviews: A Case Study of Multichannel Retailers in the Fashion Industry. Ph.D. Thesis.

[2] Hu N., Bose I., Koh N.S., Liu L. (2012). Manipulation of online reviews An analysis of ratings, readability, and sentiments. Decis. Support Syst..

[3] Hu N., Liu L., Sambamurthy V. (2011). Fraud detection in online consumer reviews. Decis. Support Syst..

[4] Li C., Wang P., Wang C., Zhang L., Liu Z., Ye Q., Xu Y., Huang F., Zhang X., Yu P.S. (2025). Loki’s Dance of Illusions: A Comprehensive Survey of Hallucination in Large Language Models. arXiv.

[5] Akhtar N., Akhtar M.N., Siddiqi U.I., Riaz M., Zhuang W. (2020). Unveiling the effects of figurative meanings in manipulated online hotel reviews on consumers’ behavioral intentions. Asia Pac. J. Mark. Logist..

[6] Anderson E.T., Simester D.I. (2014). Reviews without a purchase: Low ratings, loyal customers, and deception. J. Mark. Res..

[7] Al Wahshat H., Abu-ulbeh W., Yusoff M.H., Zakaria M.D., Hamzah W.M.A.F.W., NP S. The detection of e-commerce manipulated reviews using GPT-4. Proceedings of the 2023 International Conference on Computer Science and Emerging Technologies (CSET).

[8] Aris T.N.M., Raman K.A., Shahrel A., Nazri A. (2025). Fuzzyfakeroberta: Fake Review Identification in E-Commerce Platform. J. Theor. Appl. Inf. Technol..

[9] Görnitz N., Kloft M., Rieck K., Brefeld U. (2013). Toward supervised anomaly detection. J. Artif. Intell. Res..

[10] Liu S., Li C., Qiu J., Zhang X., Huang F., Zhang L., Hei Y., Yu P.S. (2025). The Scales of Justitia: A Comprehensive Survey on Safety Evaluation of LLMs. arXiv.

[11] Zhao S., Hong X., Yang J., Zhao Y., Ding G. (2023). Toward label-efficient emotion and sentiment analysis. Proc. IEEE.

[12] Naidu G., Zuva T., Sibanda E. A Comprehensive Review of Multi-Domain Sentiment Analysis: Techniques, Models and Future Directions. Proceedings of the International Conference on Intelligent and Innovative Computing Applications.

[13] Chen S., Long X., Fan J., Jin G. (2026). A causal inference-based root cause analysis framework using multi-modal data in large-complex system. Reliab. Eng. Syst. Saf..

[14] Tan Z., Jiang M. (2023). User modeling in the era of large language models: Current research and future directions. arXiv.

[15] Hou Y., Li J., He Z., Yan A., Chen X., McAuley J. (2024). Bridging Language and Items for Retrieval and Recommendation. arXiv.

[16] McAuley J., Contributors H.F. (2023). Amazon Reviews 2023. https://huggingface.co/datasets/McAuley-Lab/Amazon-Reviews-2023.

[17] Yin M., Wan M., Lin Z., Jiang J. (2026). Moralization-aware identity fusion for detecting violent radicalization in social media. Inf. Process. Manag..

[18] Thilini Jayasinghe J., Dassanayaka S. (2025). Detecting deception: Employing deep neural networks for fraudulent review detection on Amazon. Neural Comput. Appl..

[19] D’Souza D.J., Uday Kumar Reddy K. (2019). Anomaly detection for big data using efficient techniques: A review. Artificial Intelligence and Data Engineering.

[20] Xu X., Liu H., Yao M. (2019). Recent progress of anomaly detection. Complexity.

[21] Chen Y., He S., Wang B., Feng Z., Zhu G., Tian Z. (2024). A Verifiable Privacy-Preserving Federated Learning Framework Against Collusion Attacks. IEEE Trans. Mob. Comput..

[22] He L., Wang X., Chen H., Xu G. (2022). Online spam review detection: A survey of literature. Hum.-Centric Intell. Syst..

[23] Liu M., Zhang H., Xu Z., Ding K. (2024). The fusion of fuzzy theories and natural language processing: A state-of-the-art survey. Appl. Soft Comput..

[24] Li Y., Goodge A., Liu F., Foo C.S. Promptad: Zero-shot anomaly detection using text prompts. Proceedings of the IEEE/CVF Winter Conference on Applications of Computer Vision.

[25] Beltagy I., Cohan A., Logan IV R., Min S., Singh S. Zero-and few-shot nlp with pretrained language models. Proceedings of the 60th Annual Meeting of the Association for Computational Linguistics: Tutorial Abstracts.

[26] Yang S., Du Y., Liu J., Li X., Chen X., Gao H., Xie C., Li Y. (2024). Few-shot multi-domain text intent classification with Dynamic Balance Domain Adaptation Meta-learning. Expert Syst. Appl..

[27] Yadav G., Sharma H., Sharma D., Rai A.K. A Study on Deep Learning Based Anomaly Detection Technique in Natural Language Processing Textual Data. Proceedings of the 2024 2nd International Conference on Advances in Computation, Communication and Information Technology (ICAICCIT).

[28] Abdullah N.A., Feizollah A., Sulaiman A., Anuar N.B. (2019). Challenges and recommended solutions in multi-source and multi-domain sentiment analysis. IEEE Access.

[29] Ferdush J., Kamruzzaman J., Karmakar G., Gondal I., Das R. (2025). Cross-Domain Fake News Detection Through Fusion of Evidence from Multiple Social Media Platforms. Future Internet.

[30] Kaliyar R.K., Goswami A., Narang P. (2021). FakeBERT: Fake news detection in social media with a BERT-based deep learning approach. Multimed. Tools Appl..

[31] Glazkova A., Glazkov M., Trifonov T. (2021). g2tmn at constraint@ aaai2021: Exploiting CT-BERT and ensembling learning for COVID-19 fake news detection. Combating Online Hostile Posts in Regional Languages during Emergency Situation.

[32] Catelli R., Bevilacqua L., Mariniello N., Di Carlo V.S., Magaldi M., Fujita H., De Pietro G., Esposito M. (2023). A new Italian Cultural Heritage data set: Detecting fake reviews with BERT and ELECTRA leveraging the sentiment. IEEE Access.

[33] Bilal M., Almazroi A.A. (2022). Effectiveness of fine-tuned BERT model in classification of helpful and unhelpful online customer reviews. Electron. Commer. Res..

[34] Gambo I., Massenon R., Lin C.C., Ogundokun R.O., Agarwal S., Pak W. (2024). Enhancing user trust and interpretability in AI-driven feature request detection for mobile app reviews: An explainable approach. IEEE Access.

[35] Ayemowa M.O., Ibrahim R., Bena Y.A. (2024). A systematic review of the literature on deep learning approaches for cross-domain recommender systems. Decis. Anal. J..

[36] Wang Y., Luo Z. Enhance multi-domain sentiment analysis of review texts through prompting strategies. Proceedings of the 2023 International Conference on High Performance Big Data and Intelligent Systems (HDIS).

[37] Olteanu M., Rossi F., Yger F. (2023). Meta-survey on outlier and anomaly detection. Neurocomputing.

[38] Chung H.W., Hou L., Longpre S., Zoph B., Tay Y., Fedus W., Li Y., Wang X., Dehghani M., Brahma S. (2024). Scaling instruction-finetuned language models. J. Mach. Learn. Res..

[39] Salminen J., Kandpal C., Kamel A.M., Jung S.G., Jansen B.J. (2022). Amazon Fake Reviews Dataset. Dataset of Fake vs. Real Product Reviews Generated for Research; Originally Described in “Creating and Detecting Fake Reviews of Online Products”. https://osf.io/tyue9/.

[40] Team H.F.D. (2015). Yelp Review Full Dataset. Accessed on 19 June 2025. 650k Training Reviews + 50k Test Reviews in 5-Class Sentiment Classification Format. https://huggingface.co/datasets/Yelp/yelp_review_full.

[41] Yafooz W.M., Alsaeedi A., Alluhaibi R., Abdel-Hamid M.E. (2022). Enhancing multi-class web video categorization model using machine and deep learning approaches. Int. J. Electr. Comput. Eng. (IJECE).

[42] Alhejaili R., Alhazmi E.S., Alsaeedi A., Yafooz W.M.S. Sentiment analysis of the COVID-19 vaccine for Arabic tweets using machine learning. Proceedings of the 2021 9th International Conference on Reliability, Infocom Technologies and Optimization (Trends and Future Directions) (ICRITO).

